# B/F/TAF in people with both HIV-1 and hepatitis B: outcomes from the ALLIANCE open-label extension phase

**DOI:** 10.1186/s12981-026-00891-4

**Published:** 2026-07-16

**Authors:** Anchalee Avihingsanon, Chee Loon Leong, Chien-Ching Hung, Sasisopin Kiertiburanakul, Man-Po Lee, Michelle L. D’Antoni, Sharline Madera, Hongyuan Wang, Jason T. Hindman, Taisheng Li

**Affiliations:** 1 HIV-NAT, Thai Red Cross AIDS and Infectious Diseases Research Centre, Bangkok, Thailand; 2 Center of Excellence in Tuberculosis, Faculty of Medicine, Chulalongkorn University, Bangkok, Thailand; 3 Department of Medicine, Kuala Lumpur General Hospital, Kuala Lumpur, Malaysia; 4 Department of Internal Medicine, National Taiwan University Hospital Yunlin Branch, Yunlin, Taiwan; 5 Department of Internal Medicine, National Taiwan University Hospital and National Taiwan University College of Medicine, Taipei, Taiwan; 6 Department of Tropical Medicine and Parasitology, National Taiwan University College of Medicine, Taipei, Taiwan; 7 Faculty of Medicine Ramathibodi Hospital, Mahidol University, Bangkok, Thailand; 8 Department of Medicine, Queen Elizabeth Hospital, Kowloon, Hong Kong China; 9 Clinical Virology, Gilead Sciences, Inc., Foster City, CA USA; 10 Clinical Development, Gilead Sciences, Inc., Foster City, CA USA; 11 Biostatistics, Gilead Sciences, Inc., Foster City, CA USA; 12 Department of Infectious Disease, Peking Union Medical College Hospital, Beijing, China

**Keywords:** Chronic hepatitis B, Tenofovir, Functional cure, HIV, HBV, B/F/TAF, ALLIANCE, Clinicaltrial, Switch

## Abstract

**Background:**

Data on bictegravir/emtricitabine/tenofovir alafenamide (B/F/TAF) in people with both HIV-1 and HBV are limited, particularly for treatment-experienced people.

**Methods:**

ALLIANCE was a randomized, double-blind, active-controlled, phase 3 study of B/F/TAF versus dolutegravir (DTG) + emtricitabine/tenofovir disoproxil fumarate (F/TDF) in treatment-naïve adults with HIV-1 RNA ≥ 500 copies/mL and HBV DNA ≥ 2000 IU/mL. Participants received continuous B/F/TAF through 144 weeks (≥ 96 weeks of blinded treatment plus 48-week optional open-label extension [OLE]) or switched to B/F/TAF after ≥ 96 weeks of DTG + F/TDF through 48 weeks of OLE. Here we report outcomes from the OLE.

**Results:**

121 participants were included in the continuous B/F/TAF group; 89 in the switch group. Median B/F/TAF exposure was 186.4 and 48 weeks, respectively. HIV-1 and HBV suppression rates (missing = excluded) were maintained in both groups (B/F/TAF Week 144: 99.0% and 80.2%; switch OLE Week 48: 95.4% and 86.6%, respectively). Both groups had improved alanine aminotransferase normalization. HBV envelope antigen loss and seroconversion occurred in 44.8% and 29.3%, respectively, of participants receiving continuous B/F/TAF (Week 144) and 17.0% and 12.8% in the switch group (OLE Week 48). HBV surface antigen loss and seroconversion occurred in 22.5% and 15.5% (continuous B/F/TAF), and 4.3% and 0% (switch group) of participants, respectively. One participant (continuous B/F/TAF) discontinued due to study drug–unrelated treatment-emergent adverse event. No treatment-emergent resistance was reported.

**Conclusion:**

B/F/TAF was effective in HIV-1 and HBV suppression and well tolerated in treatment-naïve individuals for ≤ 3 years and ≤ 1 year after switching from a TDF-based regimen.

*Trial registration* ClinicalTrials.gov NCT03547908 (first registration 2018-05-24).

**Supplementary Information:**

The online version contains supplementary material available at https://doi.org/10.1186/s12981-026-00891-4.

## Background

An estimated 3.1 million people (8.4%) live with both HIV-1 and HBV worldwide [1]. The burden of coinfection varies by geographic region, with the highest rates in West and Central Africa, the Middle East and North Africa, and Asia and the Pacific.

Although antiretroviral therapies (ARTs) with dual activity against HBV and HIV-1 have improved control of HBV viremia and reduced the risk of HBV drug resistance, people with both HIV-1 and HBV have poorer outcomes (including an increased risk of liver disease and mortality) and higher healthcare utilization compared to those with HBV only [2–5].

People coinfected with HIV‑1 and HBV generally require long‑term antiviral treatment. Optimal ART aims to achieve and maintain long-term virologic suppression of HIV-1 and HBV with high tolerability, minimal risk of emergent resistance, low drug–drug interactions, and a low pill burden [6, 7]. ARTs based on tenofovir alafenamide (TAF) or tenofovir disoproxil fumarate (TDF) are recommended as an initial regimen for most adults and adolescents with HIV-1 and HBV [6–8].

Bictegravir/emtricitabine/tenofovir alafenamide (B/F/TAF) is a single-tablet regimen with demonstrated efficacy, safety, and high barrier to resistance in clinical trials and routine clinical practice [9–17]. B/F/TAF is recommended by HIV international guidelines for treatment-naïve (TN) and treatment-experienced (TE) people with HIV-1 [6–8].

The ALLIANCE study evaluated the efficacy and safety of B/F/TAF as initial therapy in people with both HIV-1 and HBV, who were TN for both viruses at baseline [18]. The results of the coprimary endpoints demonstrated that B/F/TAF was noninferior to dolutegravir plus emtricitabine/TDF (DTG + F/TDF) at achieving HIV-1 RNA suppression, and superior at achieving HBV DNA suppression at Week 48 in TN adults [18]. High rates of HIV-1 and HBV suppression were observed at Week 96 in both groups [18]. After the last participant completed the Week 96 visit of the blinded phase, all participants had the option to enter an open-label extension (OLE) phase to receive open-label B/F/TAF for 48 weeks.

Here, we present (1) cumulative efficacy and safety outcomes with B/F/TAF through Week 144 (≥ 96 weeks in the blinded phase plus ≤ 48 weeks of OLE) in TN participants initially randomized to B/F/TAF (“continuous B/F/TAF group”) and (2) efficacy and safety during the 48-week OLE in TE participants who switched to B/F/TAF from DTG + F/TDF at the start of the OLE phase (“switch group”).

## Methods

### Study design and participants

ALLIANCE (NCT03547908) was a randomized, double-blind, phase 3, multicenter study conducted at 46 outpatient centers in China, Dominican Republic, Hong Kong, Japan, Malaysia, South Korea, Spain, Taiwan, Thailand, Turkey, and the USA. Detailed methods have been described previously [18]. People with both HIV-1 and HBV who were TN for both viruses, aged ≥ 18 years with HIV-1 RNA ≥ 500 copies/mL, HBV DNA ≥ 2000 IU/mL, and confirmed sensitivity to emtricitabine and tenofovir were eligible. Participants were recruited between 30 May 2018 and 30 March 2021 (for site-specific dates see Table S1). All participants provided written informed consent. Informed consent was obtained using Institutional Review Board/Independent Ethics Committee‑approved consent forms in accordance with International Council for Harmonisation of Technical Requirements for Pharmaceuticals for Human Use guidelines for Good Clinical Practice and local regulatory requirements. At international study sites, consent materials were translated and qualified interpreters were provided as needed to ensure participant understanding. The study was conducted in accordance with the principles of the Declaration of Helsinki. The study protocol was approved by independent review boards and ethics committees (Table S2).

### Study treatments

Eligible participants were randomized 1:1 to receive B/F/TAF (50/200/25 mg) or DTG + F/TDF (50 mg + 200/300 mg), and matched placebo once daily for 96 weeks (Fig S1). Randomization was stratified by HBV envelope antigen (HBeAg) status (positive vs negative), HBV DNA level (< 8 vs ≥ 8 log_10_ IU/mL), and CD4 cell count (< 50 vs ≥ 50 cells/μL) at screening. All participants continued to take study drugs until the last subject completed the Week 96 visit of the blinded phase. On completion of the double-blinded phase, participants in both treatment groups were given the option to receive B/F/TAF for 48 weeks in the OLE phase. Entry into the OLE and receipt of B/F/TAF were optional and offered per protocol; non-participation primarily reflected participant choice or site/country non-participation rather than safety- or efficacy-driven recommendations.

### Procedures

Safety and efficacy assessments through Week 96 have been described previously [18]. Participants who entered the OLE phase continued scheduled visits every 12 weeks. Levels of HIV-1 RNA, HBV DNA, and alanine aminotransferase (ALT), CD4 cell count, and safety were assessed at every visit. HBV serology (HBeAg and HBV surface antigen [HBsAg]) was assessed every 48 weeks in the OLE phase. Adverse events (AEs) were coded using the Medical Dictionary for Regulatory Activities (version 24.1 for Week 48 of the blinded phase and version 25.1 thereafter).

All participants underwent protease and reverse transcriptase HIV‑1 genotypic analysis at screening to confirm susceptibility to emtricitabine and tenofovir per the inclusion criteria [18]. In addition, HIV-1 genotypic and phenotypic analyses [18] of protease,reverse transcriptase, and integrase were performed on plasma samples from participants with confirmed HIV-1 virologic rebound (defined as HIV-1 RNA ≥ 50 copies/mL after achieving HIV-1 < 50 copies/mL or > 1 log_10_ increase in HIV-1 RNA from nadir, and HIV-1 RNA ≥ 200 copies/mL at subsequent visit) or HIV-1 RNA ≥ 200 copies/mL at the last on-treatment visit. Participants who subsequently did not resuppress on study drugs were included in the final resistance analysis population.

Serum samples for potential sequence analysis of HBV polymerase/reverse transcriptase (pol/RT) were collected at every visit. Sequencing analysis of the HBV pol/RT was completed for all participants who maintained viremia (HBV DNA ≥ 69 IU/mL) at Week 48, Week 96, and at early study drug discontinuation visit, and for those with virologic breakthrough. Virologic breakthrough was defined as two consecutive HBV DNA values ≥ 29 IU/mL after previously achieving < 29 IU/mL, or two consecutive ≥ 1.0 log_10_ increases in HBV DNA from nadir for those who did not achieve < 29 IU/mL.

### Outcomes

The coprimary efficacy endpoints of proportion of participants with HIV-1 RNA < 50 copies/mL and proportion of participants with HBV DNA < 29 IU/mL at Week 48, and secondary endpoints at Week 96, have been reported previously [18]. The endpoints described here are from B/F/TAF initiation (1) through Week 144 in the continuous B/F/TAF group and (2) through Week 48 of OLE in the switch group, and include the proportion of participants with HIV-1 RNA < 50 copies/mL, HBV DNA < 29 IU/mL, ALT normalization (a change in ALT level from above the upper limit of normal [ULN; per 2018 American Association for the Study of Liver Disease (AASLD) criteria [19]] at baseline to ALT ≤ ULN), normal ALT level (≤ ULN; 25 U/L for females and 35 U/L for males, per 2018 AASLD criteria [19]), HBeAg and HBsAg loss and seroconversion, and AEs. Changes in fasting glucose and lipid levels, body weight, estimated glomerular filtration rate by Cockcroft-Gault equation (eGFR_CG_), and treatment-emergent HIV-1 or HBV resistance are also reported.

ALT flare was defined as confirmed serum ALT > 2 × Day 1 value and > 10 × ULN, with or without associated symptoms.

### Statistical analysis

Baseline was considered to be B/F/TAF initiation – i.e., the start of the blinded phase (study entry) for the continuous B/F/TAF group, and the start of B/F/TAF in the OLE phase for the switch group. HIV-1 and HBV suppression, CD4 cell counts, and HIV-1 and HBV resistance were assessed in the all B/F/TAF full analysis set, which included all randomized participants who received at least one dose of study drug and had at least one post-baseline HIV-1 RNA or HBV DNA result while on study drug. ALT normalization was assessed for all participants in the all B/F/TAF full analysis set with baseline ALT > ULN. HBeAg and HBsAg loss/seroconversion were assessed in the all B/F/TAF serologically evaluable full analysis set, which included all participants in the all B/F/TAF full analysis set who were HBeAg/HBsAg positive and HBV envelope/surface antibody (HBeAb/HBsAb) negative or had missing data at baseline.

All efficacy outcome analyses were based on on-treatment data (i.e., up to 1 day after the last dose date of study drug). Data were reported as either the number and proportion of participants achieving each outcome, or as the proportion of participants achieving each outcome with associated 95% confidence intervals. HIV-1 RNA, HBV DNA, ALT normalization, and HBeAg/HBsAg loss/seroconversion were analyzed using the missing = excluded (M = E) method, where participants with missing data at a given time point were excluded from both the numerator and denominator in the computation of percentages. The 95% confidence interval for the proportion of participants with HIV-1 RNA < 50 copies/mL or HBV DNA < 29 IU/mL was constructed using the Clopper-Pearson exact method.

Demographic and baseline characteristics, and changes from baseline in CD4 cell count were summarized using standard descriptive methods (e.g., number of participants [proportion] or median [quartile 1, quartile 3 (Q1, Q3)] values).

Safety outcomes were analyzed in the safety analysis set, which included all participants randomized into the study who had received at least one dose of study drug. All the data collected up to 30 days after participants permanently discontinued their study drug were included in the analysis unless specified otherwise. Data were reported as the number and proportion of participants experiencing each outcome, or as median (Q1, Q3) values.

The continuous B/F/TAF and switch groups were not directly comparable due to differences in participant populations and duration of treatment. Therefore, formal statistical comparisons between treatment groups were not performed.

## Results

### Participant disposition

Of 121 participants initially randomized to and treated with B/F/TAF (continuous B/F/TAF group; Fig S2), 111 completed B/F/TAF treatment in the blinded phase. Of these, 95 entered the OLE phase; 90 completed OLE and 5 discontinued B/F/TAF (due to death [n = 1], nonadherence to study drug [n = 1], and loss to follow-up [n = 3]). Median (Q1, Q3) exposure to B/F/TAF was 186.4 (160.1, 222.3) weeks in the continuous B/F/TAF group.

Of 110 participants who completed DTG + F/TDF treatment in the blinded phase, 89 switched to B/F/TAF at the start of the OLE phase (switch group; Fig S2); 88 participants completed OLE and 1 discontinued B/F/TAF (due to loss to follow-up). Median (Q1, Q3) exposure to B/F/TAF was 48.0 (47.9, 48.6) weeks in the switch group.

### Baseline demographics and clinical characteristics

Baseline characteristics are presented in Table 1. Most participants were Asian males.

**Table 1:** Demographics and disease characteristics at B/F/TAF initiation

	Continuous B/F/TAF (n = 121)	DTG + F/TDF → B/F/TAF switch (n = 89)^a^
Age, median (Q1, Q3), years	31 (27, 39)	34 (28, 39)
Male sex at birth, n (%)	112 (93)	87 (98)
Race, n (%)		
Asian	108 (89)	84 (94)
White	10 (8)	0
Black	2 (2)	4 (4)
Other	1 (1)	1 (1)
Hispanic or Latino ethnicity, n (%)	7 (6)	5 (6)
HIV-1 RNA^b^		
Median (Q1, Q3), log_10_ copies/mL	4.66 (4.22, 5.12)	1.28 (1.28, 1.28)
≥ 50 copies/mL, n (%)	119 (100)	3 (3)
≥ 100,000 copies/mL, n (%)	38 (31)	0
CD4 count, median (Q1, Q3), cells/µL	245 (127,383)	497 (320, 617)
Mode of HIV-1 transmission, n (%)		
Homosexual sex	88 (73)	74 (83)
Heterosexual sex	29 (24)	15 (17)
Intravenous drug use	0	3 (3)
Unknown	4 (3)	2 (2)
Other	2 (2)	0
Body weight, median (Q1, Q3), kg	63.7 (57.0, 73.9)	67 (59, 75)
BMI, median (Q1, Q3), kg/m^2^	22.2 (19.9, 24.7)	22 (20, 26)
ALT level, median (Q1, Q3), U/L	34 (23, 60)	26 (18, 38)
ALT level > ULN, n (%)^c^	60 (50)	27 (30)
HBV DNA		
Median (Q1, Q3), log_10_ IU/mL	7.96 (6.52, 8.38)	0.95 (0.95, 1.20)
≥ 29 IU/mL, n (%)	118 (99)	15 (17)
≥ 8 log_10_ IU/mL, n (%)	60 (50)	0
Positive for HBeAg, n (%)	92 (76)	52 (58)
Positive for HBsAg, n (%)	121 (100)	77 (87)
HBV genotype, n (%)		
A	7 (6)	7 (9)
B	21 (19)	15 (19)
C	63 (56)	49 (61)
D	15 (13)	8 (10)
F	3 (3)	0
Mixed	3 (3)	1 (1)
Missing	9	9
Mode of HBV transmission,^d^ n (%)		
Vertical transmission	29 (24)	14 (16)
Contact with infected individual (other than vertical transmission)	23 (19)	16 (18)
Blood product transfusion	1 (1)	0
Injection drug use	1 (1)	0
Unknown	63 (52)	59 (66)
Other	4 (3)	0
Time since HBV diagnosis, median (Q1, Q3), years^e^	0.2 (0.1, 4.1)	3.6 (2.6, 4.8)

The continuous B/F/TAF and switch groups are not directly comparable due to different participant populations and duration of treatment

^a^For the switch group, demographics and disease characteristics are presented at OLE baseline

^b^HIV-1 RNA values below the lower limit of quantification were imputed per the statistical analysis plan

^c^Based on the 2018 American Association for the Study of Liver Diseases criteria; ULN is 25 U/L for females and 35 U/L for males

^d^Mode of transmission was self-reported in accordance with protocol-specified data collection at baseline

^e^Self-reported or based on examination/medical records

ALT, alanine aminotransferase; B/F/TAF, bictegravir/emtricitabine/tenofovir alafenamide; CD, cluster of differentiation; DTG, dolutegravir, F/TDF, emtricitabine/tenofovir disoproxil fumarate; HBeAg, HBV envelope antigen; HBsAg, HBV surface antigen; HBV, hepatitis B virus; OLE, open-label extension; Q, quartile; ULN, upper limit of normal

Participants in the continuous B/F/TAF group were TN for both HIV-1 and HBV at baseline (study entry). Thirty-one percent (38/121) of participants had HIV-1 RNA ≥ 100,000 copies/mL, 50% (60/121) had HBV DNA ≥ 8 log_10_ IU/mL, and the median (Q1, Q3) CD4 cell count was 245 (127, 383) cells/µL. Half of participants in this group had ALT > ULN, 76% (92/121) and 100% (121/121) were positive for HBeAg and HBsAg, respectively. Participants in the switch group had already received ≥ 96 weeks of treatment with DTG + F/TDF prior to switching to B/F/TAF at the start of OLE. At B/F/TAF initiation, 3 out of 89 (3%) participants had HIV-1 RNA ≥ 50 copies/mL (2 had viral load between 50 and < 200 copies/mL, and 1 had ≥ 1000 copies/mL) and 15 (17%) participants had HBV DNA ≥ 29 IU/mL (9 had viral load between 29 and < 69 IU/mL and 6 had ≥ 69 IU/mL); the median (Q1, Q3) CD4 cell count was 497 (320, 617) cells/µL. Thirty percent (27/89) of participants had ALT > ULN; 58% (52/89) and 87% (77/89) were positive for HBeAg or HBsAg, respectively.

### Virologic and immunologic outcomes

**Fig. 1:**
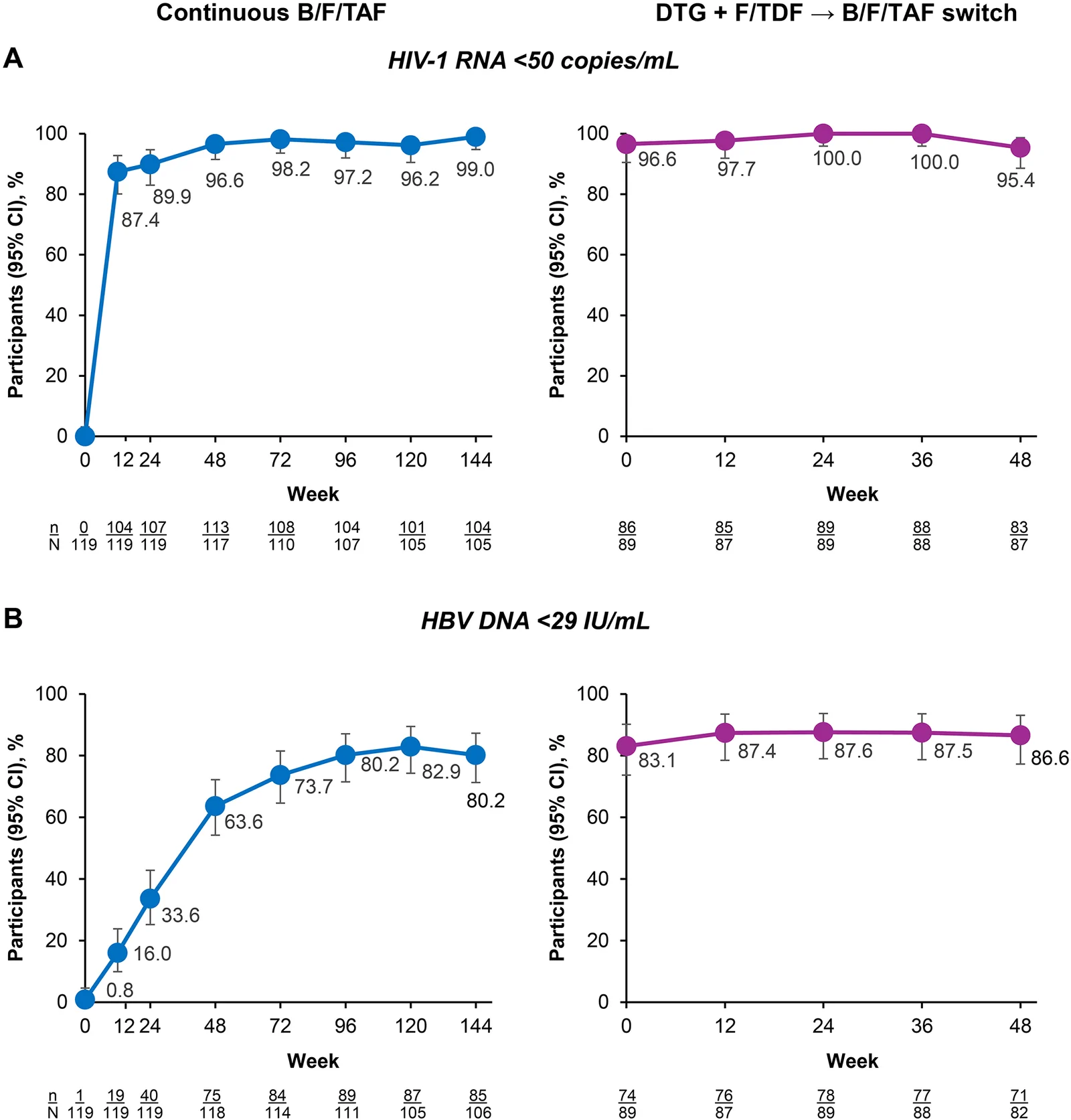
Virologic suppression over time from B/F/TAF initiation (M = E analysis). HIV-1 and HBV suppression was assessed in the all B/F/TAF full analysis set with available data. The continuous B/F/TAF and switch groups are not directly comparable due to different participant populations and duration of treatment. B/F/TAF, bictegravir/emtricitabine/tenofovir alafenamide; CI, confidence interval; DTG, dolutegravir, F/TDF, emtricitabine/tenofovir disoproxil fumarate; M = E, missing = excluded

HIV-1 and HBVsuppression (HIV-1 RNA < 50 copies/mL and HBV DNA < 29 IU/mL, respectively) rates by M = E analysis were high in both groups (Fig. 1).

In the continuous B/F/TAF group, most participants had achieved HIV-1 suppression at 12 weeks (87.4%; 104/119), and 99.0% (104/105) had HIV-1 suppression at Week 144. HBV suppression increased over the first 96 weeks of treatment and was maintained through end of OLE, with high rates of suppression observed at Week 144 (80.2% [85/106]). Median (Q1, Q3) CD4 cell count increased from baseline by 292 (211, 457) cells/µL at OLE Week 48 (Fig S3) and by 277 (150, 398) cells/µL at Week 144.

In the switch group, the proportion of participants with HIV-1 suppression was high at entry to the OLE phase (prior to B/F/TAF initiation; 96.6% [86/89]), and this was maintained through the end of OLE, with 95.4% (83/87 participants) having HIV-1 suppression after 48 weeks of B/F/TAF treatment. Similarly, the proportion of participants with HBV suppression was high at B/F/TAF initiation (83.1% [74/89]), and this was maintained through the end of OLE, with 86.6% (71/82 participants) having HBV suppression after 48 weeks of B/F/TAF treatment. Median (Q1, Q3) CD4 cell count increased from study baseline by 248 (162, 410) cells/µL to OLE Week 48 (Fig S3), including by 29 (− 44, 107) cells/µL after 48 weeks of B/F/TAF in the OLE.

### HBV outcomes

**Fig. 2:**
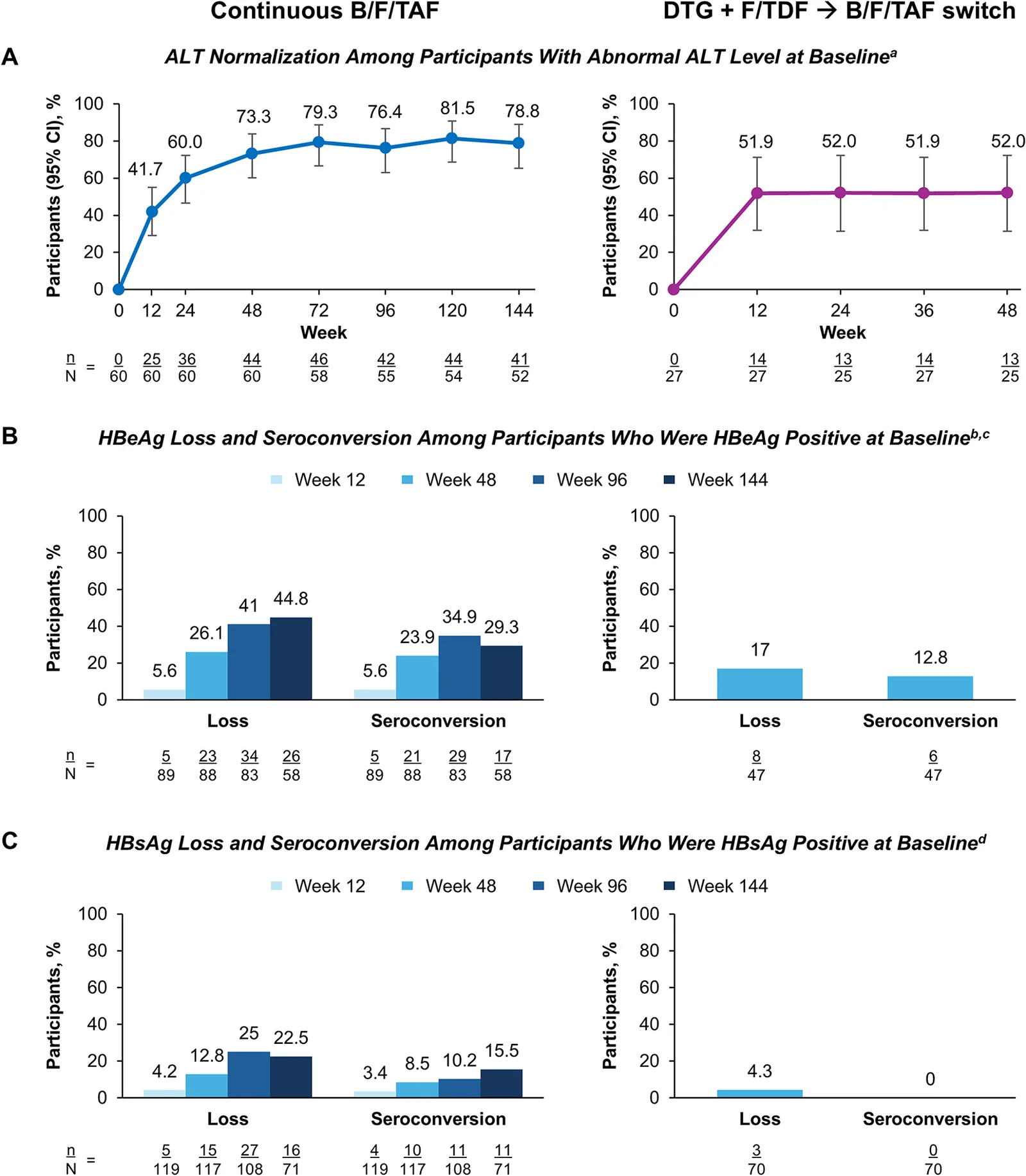
HBV outcomes over time from B/F/TAF initiation (M = E analysis). The continuous B/F/TAF and switch groups are not directly comparable due to differences in participant populations and duration of treatment. ALT normalization was assessed in the all B/F/TAF full analysis set with baseline ALT level > ULN. The all B/F/TAF full analysis set included all randomized participants who received ≥ 1 dose of study drug and had ≥ 1 post-baseline HIV-1 RNA or HBV DNA result while on study drug. HBsAg and HBeAg loss/seroconversion were assessed in the all B/F/TAF serologically evaluable full analysis set, defined as all participants in the all B/F/TAF full analysis set who were HBeAg or HBsAg positive and HBeAb or HBsAb negative or had data missing at baseline. The denominator is the number of participants with non-missing data at each endpoint. Differences in denominators are due to not all countries participating in the OLE and missing data. ^a^In the switch group, baseline is OLE baseline, i.e., at B/F/TAF initiation. ^b^At Week 144 in the continuous B/F/TAF group, 8.6% (5/58) of participants had new HBeAg loss and 3.4% (2/58) had new HBeAg seroconversion (i.e., no HBeAg loss at Week 96). ^c^At OLE Week 48 in the switch group, 17.0% (8/47) of participants had new HBeAg loss and 12.8% (6/47) had new HBeAg seroconversion. ^d^At Week 144 in the continuous B/F/TAF group, 1.4% (1/71) of participants had new HBsAg loss and 5.6% (4/71) had new HBsAg seroconversion (i.e., had no HBsAg loss at Week 96). ALT, alanine aminotransferase; B/F/TAF, bictegravir/emtricitabine/tenofovir alafenamide; CI, confidence interval; DTG, dolutegravir, F/TDF, emtricitabine/tenofovir disoproxil fumarate; HBeAb, HBV envelope antibody; HBeAg, HBV envelope antigen; HBsAb, HBV surface antibody; HBsAg, HBV surface antigen; HBV, hepatitis B virus; M = E, missing = excluded; OLE, open-label extension; ULN, upper limit of normal

In the continuous B/F/TAF group, the proportion of participants with ALT normalization (i.e., a change in ALT level from above the ULN to ≤ ULN) increased gradually up to Week 72 and was maintained thereafter, with a rate of 78.8% (41/52) at Week 144 (Fig. 2A). Similarly, the proportion of participants with normal ALT level increased to 77.9% (88/113) by Week 72, which was generally maintained through Week 144, when 80.2% (85/106) of participants had normal ALT level (Fig S4). Among participants with a positive HBeAg test at baseline, 44.8% (26/58) had HBeAg loss and 29.3% (17/58) had HBeAg seroconversion at Week 144 (Fig. 2B). Of these participants, 8.6% (5/58) had new HBeAg loss and 3.4% (2/58) had new HBeAg seroconversion at Week 144 (i.e., had no HBeAg loss or seroconversion at Week 96, respectively). Among participants with a positive HBsAg test at baseline, 22.5% (16/71) had HBsAg loss and 15.5% (11/71) had HBsAg seroconversion at Week 144 (Fig. 2C). Of these participants, 1.4% (1/71) had new HBsAg loss and 5.6% (4/71) had new HBsAg seroconversion at Week 144 (i.e., had no HBsAg loss or seroconversion at Week 96, respectively).

In the switch group, 51.9% (14/27) of participants with abnormal ALT level at B/F/TAF initiation achieved ALT normalization after 12 weeks of B/F/TAF treatment, with this proportion remaining stable to OLE Week 48 (Fig. 2A). The proportion of participants with normal ALT level increased by 12.2% in the first 12 weeks of B/F/TAF treatment and then remained stable, with 79.3% (65/82) of participants having normal ALT level at OLE Week 48 (Fig S4). During 48 weeks of OLE, 17.0% (8/47) of participants had new HBeAg loss and 12.8% (6/47) had new HBeAg seroconversion (Fig. 2B); 4.3% (3/70) of participants had new HBsAg loss, and none had new HBsAg seroconversion (Fig. 2C).

### Safety

While most participants experienced at least one AE, the majority were Grade 1 or 2 in severity and not considered to be study drug related (Table 2). None of the study drug–related events were Grade 4.

**Table 2:** Cumulative adverse events and laboratory abnormalities from B/F/TAF initiation through end of the OLE phase

	Continuous B/F/TAF (n = 121)^a^	DTG + F/TDF → B/F/TAF switch (n = 89)
Any AEs, n (%)	117 (97)	66 (74)
Grade 3 or 4 AEs	26 (21)	6 (7)
Serious AEs	20 (17)	2 (2)
Study drug–related AEs, n (%)	39 (32)	17 (19)
Grade 3 or 4 AEs	8 (7)^b^	3 (3)^c^
Serious AEs	1 (1)^d^	0
Study drug–related AEs (≥ 3% of participants in either group), n (%)		
Weight gain^e^	13 (11)	8 (9)
ALT increase	4 (3)	2 (2)
Dyslipidemia	4 (3)	1 (1)
Headache	4 (3)	0
LDL cholesterol increase	1 (1)	3 (3)
Study drug discontinuation due to AEs, n (%)	1 (1)^f^	0
Death, n (%)	3 (2)^g^	0
Any laboratory abnormalities, n (%)^h^		
Any Grade 3 or 4	116 (97)	79 (89)
Grade 3 or 4 occurring in ≥ 10% of participants in either group	54 (45)	7 (8)
ALT increase	27 (23)	0
AST increase	16 (13)	0

The continuous B/F/TAF and switch groups are not directly comparable due to different participant populations and duration of treatment

^a^For the continuous B/F/TAF group, data are presented from the blind phase baseline and for the switch group, from OLE baseline

^b^All events were Grade 3: abnormal weight gain (n = 2), ALT increase, cryptococcal meningitis, hypomagnesemia, major depression, serum creatinine increase, serum triglycerides increase, and weight increase (n = 1 each; hypomagnesemia and serum triglycerides increase in the same participant)

^c^All events were Grade 3: abnormal weight gain (n = 2) and hyperlipidemia (n = 1)

^d^Cryptococcal meningitis

^e^Includes weight increase and abnormal weight gain

^f^Due to hepatocellular carcinoma; not considered to be study drug related

^g^Includes deaths that occurred between the first dose and 30 days after the last dose. These were due to unknown cause (n = 2) and laryngeal squamous cell carcinoma (n = 1); all occurred during the blinded phase and were considered unrelated to study drug

^h ^Laboratory abnormalities were assessed in 120 participants originally assigned to B/F/TAF and 89 participants who switched from DTG + F/TDF

AE, adverse event; ALT, alanine aminotransferase; AST, aspartate aminotransferase; B/F/TAF, bictegravir/emtricitabine/tenofovir alafenamide; DTG, dolutegravir; F/TDF, emtricitabine/tenofovir disoproxil fumarate; LDL, low-density lipoprotein; OLE, open-label extension

In the continuous B/F/TAF group, the most common study drug–related AEs were weight gain (11%; 13/121), increased ALT level, dyslipidemia, and headache (each 3%; 4/121). As previously reported [18], one participant experienced a serious AE (SAE; cryptococcal meningitis) assessed by the site investigator as study drug related, 1 discontinued B/F/TAF due to an AE (hepatocellular carcinoma, considered unrelated to study drug), and 3 died (due to unknown cause [n = 2] and laryngeal squamous cell carcinoma [n = 1]; all occurred during the blinded phase and were considered unrelated to the study drug) through the end of study. Grade 3 or 4 laboratory abnormalities were reported in 45% (54/120) of participants. Increased ALT level and increased aspartate aminotransferase level were the only Grade 3 or 4 laboratory abnormalities reported in ≥ 10% of participants (23% [27/120] and 13% [16/120], respectively).

In the switch group, the most common study drug–related AEs were weight gain (9%; 8/89) and increased low-density lipoprotein (LDL) cholesterol level (3%; 3/89). There were no SAEs, B/F/TAF discontinuations due to AEs, or deaths through 48 weeks of B/F/TAF treatment. Grade 3 or 4 laboratory abnormalities were reported by 8% (7/89) of participants.

### Metabolic parameters

In the continuous B/F/TAF group, fasting glucose and lipid parameters, and body weight, remained relatively stable during follow-up, except for a small increase in total cholesterol level (Fig S5). At Week 144, median (Q1, Q3) fasting glucose was 89 (84, 97) mg/dL, LDL cholesterol 114 (93, 138) mg/dL, high-density lipoprotein (HDL) cholesterol 46 (41, 54) mg/dL, triglycerides 114 (81, 164) mg/dL, and total cholesterol to HDL cholesterol ratio 4.0 (3.4, 4.9). Median (Q1, Q3) body weight increased from baseline by 5.3 (1.6, 9.6) kg at Week 144. Median (Q1, Q3) eGFR_CG_ remained generally stable during the follow-up (Fig S6A), with minimal, non‑clinically meaningful changes from baseline (Fig S6B).

In the switch group, metabolic parameters, including body weight, remained relatively stable during follow-up, except for a small increase in total and LDL cholesterol levels (Fig S5). At Week 48 after B/F/TAF initiation, median (Q1, Q3) fasting glucose was 88 (83, 96) mg/dL, LDL cholesterol 123 (96, 140) mg/dL, HDL cholesterol 49 (41, 56) mg/dL, triglycerides 105 (70, 159) mg/dL, and total cholesterol to HDL cholesterol ratio 3.7 (3.3, 4.6). Median (Q1, Q3) body weight increased from OLE baseline by 2.0 (0.1, 4.0) kg at Week 48 after switch from DTG + F/TDF to B/F/TAF (overall median [Q1, Q3] change from start of DTG + F/TDF to OLE Week 48 was 6.0 [2.0, 10.0] kg). Median (Q1, Q3) eGFR_CG_ remained generally stable during the follow-up (Fig S6A), with minimal, non‑clinically meaningful changes from baseline in the switch group (Fig S6B).

### Resistance

Through the end of OLE, 6/119 (5.0%) participants in the continuous B/F/TAF group and 1/89 (1.1%) participants in the switch group met the criteria for HIV virologic failure and were included in the final resistance analysis population. None of these 7 participants had HIV-1 treatment-emergent resistance to study drugs. Overall, through 96 weeks of the blinded phase, 4/119 (3.36%) participants in the continuous B/F/TAF group met the criteria for HBV resistance testing. Phenotypic results were successfully generated for all except 1 participant. No HBV amino acid substitutions associated with resistance to TAF were detected through 96 weeks of B/F/TAF treatment. A detailed HIV-1 resistance analysis through Week 144 is currently underway and will be reported in a separate article. No additional HBV resistance analyses were planned. None of the participants had both HIV-1 and HBV virologic breakthrough.

## Discussion

### Interpretation

This analysis provides the first long-term data (3 years) on B/F/TAF in people TN for both HIV-1 and HBV, and the first clinical data on outcomes following a switch to B/F/TAF in a TE population (48-week follow-up).

In participants who continued B/F/TAF treatment into the OLE phase, as expected, high levels of HIV-1 RNA suppression, HBV DNA suppression, and ALT normalization were maintained through 3 years, with additional HBeAg losses and HBsAg seroconversions observed after Week 96. This further confirms the durability of dual virologic suppression with B/F/TAF.

As anticipated, in participants who switched from DTG + F/TDF to B/F/TAF, high levels of HIV-1 and HBV suppression were also maintained, with increases in rates of ALT normalization, HBeAg loss and seroconversion, and HBsAg loss during 48 weeks of OLE B/F/TAF treatment.

Treatment was well tolerated through end of study, with 1 participant discontinuing due to an AE that was deemed unrelated to study drug. Reassuringly, no new safety concerns were observed during the longer follow-up, and the safety findings were generally consistent with those previously reported with B/F/TAF [12, 14,17, 18]. Furthermore, no treatment-emergent resistance to B/F/TAF components was detected through Week 144 for HIV-1 or through Week 96 for HBV.

In the blinded phase of ALLIANCE, both B/F/TAF and DTG + F/TDF were associated with high rates of HIV-1 and HBV suppression by Week 96, and HBV outcomes (ALT normalization, HBeAg loss and seroconversion, and HBsAg loss or seroconversion) were either numerically or significantly better with B/F/TAF [18]. Consistent with these results, high rates of HIV-1 and HBV suppression were maintained over 48 weeks of OLE following the switch to B/F/TAF, and over 144 weeks in participants receiving B/F/TAF in the randomized and OLE phase.

To our knowledge, this is the first randomized study to compare TAF- and TDF-based treatment for people with HIV-1 and HBV. However, previous clinical trials and cohort studies have compared TAF and TDF in people with either HIV-1 or HBV alone [13, 20–24]. In a pooled analysis of two phase 3 studies in people with HBV monoinfection (GS-US-320-0108 and GS-US-320-0110), treatment with TAF demonstrated noninferior efficacy and an improved bone and renal safety profile compared with TDF at Weeks 48 and 96; thereafter, the proportion with HBV suppression remained relatively stable, around 90% (M = E) on open-label TAF treatment, reaching 95% at Week 384 [21, 25]. TAF has also been shown to be noninferior to TDF for HBV suppression in HBV-only studies [26, 27]. In our study, we observed a similar pattern of results in people with both HIV-1 and HBV, with most HBV suppression occurring in the first 2 years of treatment. Durable HIV-1 suppression with B/F/TAF has also been previously observed in a pooled analysis of phase 3 studies (GS-US-380-1489 and GS-US-380-1490) in people with HIV-1 after switch from a DTG-based regimen [13, 24].

In the present study, some secondary HBV outcomes improved following the switch from DTG + F/TDF to B/F/TAF; this was most noticeable for ALT normalization. At B/F/TAF initiation in the switch group (i.e., after ≥ 96 weeks of treatment with DTG + F/TDF), 30% of participants had abnormal ALT level; of these, 52% experienced ALT normalization within 12 weeks. The further normalization of ALT levels after switching from a TDF-based to a TAF-based treatment in the OLE is consistent with observations from the blinded phase of the study (72% of participants with B/F/TAF vs 57% of those with DTG + F/TDF had ALT normalization at Week 96 by missing = failure analysis [78%, vs 61% by M = E analysis; unpublished]) and from HBV-only studies [18, 20–23, 25–27]. Although the mechanism underlying higher ALT normalization with TAF remains unclear, our data add to a growing body of evidence showing that TAF is associated with a higher rate of ALT normalization than TDF [18, 20–23, 26, 27]. Adherence during the ALLIANCE study was monitored according to the protocol, and there were no differences in adherence between the TAF and TDF regimens that would explain the observed HBV outcomes [18].

A similar trend was observed for HBeAg loss and seroconversion. At B/F/TAF initiation, 58% of participants in the switch group were HBeAg positive, of whom 17% had new HBeAg loss and 13% had new HBeAg seroconversion after 48 weeks of B/F/TAF treatment. A similar increase in HBeAg loss/seroconversion after the switch was seen in studies of people with HBV only who switched from TDF to TAF [21]. The rate of HBeAg loss in our study was comparable to the 16% observed after the switch from TDF to TAF in a clinical practice cohort with chronic HBV [23]. These increased rates of ALT normalization and HBeAg loss/seroconversion, together with the improvement in renal function as evidenced by increases in eGFR after switching from DTG + F/TDF to B/F/TAF, suggest that there may be additional benefits to TAF-based therapy over TDF-based therapy for people with HIV-1 and HBV, even for individuals already receiving TDF-based ART.

One of the unexpected findings in the first 96 weeks of the ALLIANCE study was the unusually high rates of HBsAg loss and seroconversion (25.0% and 10.2%, respectively), compared with rates of up to 2.5% (HBsAg loss) and 1% (seroconversion) through 5 years in the HBV-only studies [21]. This may be due to differences between the study populations; for example in our study, participants with both acute and chronic HBV may have been enrolled (since we relied on self-reporting for mode of infection), whereas only participants with chronic HBV were enrolled in the HBV-only studies. Different study populations could also have resulted in reporting bias as well as differences in the duration of HBV infection. Rates of additional HBsAg loss in the present study were relatively low among the switch group, with no additional seroconversions; this finding likely reflects the fact that these participants had already received ≥ 96 weeks of DTG + F/TDF treatment before entering the OLE, as well as the relatively small numbers of participants in the serologically evaluable full analysis set for this group (n = 70 at Week 48). Because of the combined impact of HIV-1 and HBV on immune response, people with both viruses typically have accelerated progression of HBV infection and HBV-related liver disease complications compared with those with HBV only [28]. HBsAg loss with durable HBV suppression (functional cure) is the optimal treatment goal for chronic HBV infection; however, it is rarely observed in individuals receiving antiviral treatment for HBV alone [25]. Spontaneous HBsAg loss can occur, but is also uncommon, occurring in approximately 1% of untreated individuals with chronic HBV infection annually [27, 29]. Therefore, the high rates of HBsAg loss and seroconversion observed in our study are particularly encouraging.

Median changes in metabolic parameters were small overall. The slight increase in lipid levels in the switch group was consistent with the known lipid‑lowering effect of TDF [30]. Changes in lipid parameters during the study were monitored per the protocol and managed according to standard clinical practice. Participants in the continuous B/F/TAF group had a cumulative increase in body weight from the blinded phase baseline of 5.6 kg at OLE Week 48, while those in the switch group had a cumulative increase in body weight from the blinded phase baseline of 6.0 kg at OLE Week 48. Small non‑clinically meaningful changes in eGFR were observed after switching to B/F/TAF, consistent with previous reports of better renal safety outcomes with TAF versus TDF due to lower plasma levels of active tenofovir [20, 22, 31, 32].

### Limitations

Limitations of the study include the low number of participants and relatively short follow-up in participants who switched from DTG + F/TDF, and the high number of participants with missing HBeAg and HBsAg loss/seroconversion data. Additionally, interpretation of the results could be limited by the heterogeneous study population, as participants likely had HIV-1 and HBV prior to treatment initiation, differing HBeAg statuses (positive or negative), and either normal or elevated ALT levels. We acknowledge that the exact timing of HBV infection is not certain, as duration of HBV infection was self-reported or based on examination/medical records. Finally, not all originally randomized and treated participants entered the OLE phase, potentially introducing a selection bias.

## Conclusions

These results demonstrate the efficacy and safety of B/F/TAF in TN and TE people with both HIV-1 and HBV and further support the long-term use of B/F/TAF in people in this clinical setting.

## Supplementary Information

Supplementary Material 1.

## Data Availability

Gilead Sciences shares anonymized individual patient data upon request or as required by law or regulation with qualified external researchers based on submitted curriculum vitae and reflecting non-conflict of interest. The request proposal must also include a statistician. Approval of such requests is at Gilead Sciences’ discretion and is dependent on the nature of the request, the merit of the research proposed, the availability of the data, and the intended use of the data. Data requests should be sent to datarequest@gilead.com.

## Abbreviations

AASLD: American Association for the Study of Liver Disease
AE: Adverse event
ALT: Alanine aminotransferase
ART: Antiretroviral therapy
B/F/TAF: Bictegravir/emtricitabine/tenofovir alafenamide
DTG: Dolutegravir
eGFR_CG_: Estimated glomerular filtration rate by Cockcroft-Gault equation
F/TDF: Emtricitabine/tenofovir disoproxil fumarate
HDL: High-density lipoprotein
LDL: Low-density lipoprotein
M = E: Missing = excluded
OLE: Open-label extension
pol/RT: Polymerase/reverse transcriptase
Q: Quartile
SAE: Serious adverse event
TAF: Tenofovir alafenamide
TDF: Tenofovir disoproxil fumarate
TE: Treatment-experienced
TN: Treatment-naïve
ULN: Upper limit of normal
