# Supplementary material for: B/F/TAF in people with both HIV-1 and hepatitis B: outcomes from the ALLIANCE open-label extension phase
Source: AIDS Res Ther. 2026 Jul 16;23:85. doi: 10.1186/s12981-026-00891-4 (PMC13560426; doi:10.1186/s12981-026-00891-4)
Supplement: Supplementary file 1 [file 12981_2026_891_MOESM1_ESM.docx]

# Supplement

Table S1. Site-specific enrolment dates.

| **Site number** | **Region** | **Activation date** | **End of enrolment date** |
| --- | --- | --- | --- |
| 380-4458-5220 | China | 17-Sep-2020 | 30-Mar-2021 |
| 380-4458-15762 | China | 19-Jul-2020 | 30-Mar-2021 |
| 380-4458-23024 | China | 12-Jul-2022 | 30-Mar-2021 |
| 380-4458-15763 | China | 13-Apr-2020 | 30-Mar-2021 |
| 380-4458-15764 | China | 6-Sep-2020 | 30-Mar-2021 |
| 380-4458-15765 | China | 13-Apr-2020 | 30-Mar-2021 |
| 380-4458-15766 | China | 27-Nov-2020 | 30-Mar-2021 |
| 380-4458-15767 | China | 24-Aug-2020 | 30-Mar-2021 |
| 380-4458-15768 | China | 24-Jun-2020 | 30-Mar-2021 |
| 380-4458-10742 | China | 30-Dec-2020 | 30-Mar-2021 |
| 380-4458-11551 | China | 23-Nov-2020 | 30-Mar-2021 |
| 380-4458-28729 | Dominican Republic | 29-Sep-2023 | 30-Mar-2021 |
| 380-4458-2447 | France | 15-Nov-2018 | 9-Jul-2020 |
| 380-4458-2866 | France | 4-Oct-2018 | 30-Mar-2021 |
| 380-4458-15266 | Greece | 12-Jun-2019 | 30-Mar-2021 |
| 380-4458-15269 | Greece | 21-Mar-2019 | 30-Mar-2021 |
| 380-4458-15272 | Greece | 31-Jan-2019 | 11-Feb-2021 |
| 380-4458-6171 | Greece | 18-Feb-2019 | 30-Mar-2021 |
| 380-4458-5606 | Hong Kong | 25-Jan-2019 | 30-Mar-2021 |
| 380-4458-15234 | Hong Kong | 4-Aug-2019 | 30-Mar-2021 |
| 380-4458-15235 | Hong Kong | 31-Oct-2018 | 23-Dec-2020 |
| 380-4458-27292 | Japan | 13-Mar-2023 | 30-Mar-2021 |
| 380-4458-16753 | Japan | 21-Oct-2020 | 30-Mar-2021 |
| 380-4458-27695 | Japan | 18-Feb-2020 | 30-Mar-2021 |
| 380-4458-16964 | Japan | 16-Feb-2020 | 30-Mar-2021 |
| 380-4458-16967 | Japan | 25-Dec-2019 | 30-Mar-2021 |
| 380-4458-16965 | Japan | 14-Jan-2020 | 30-Mar-2021 |
| 380-4458-16966 | Japan | 14-May-2020 | 30-Mar-2021 |
| 380-4458-16755 | Japan | 14-Dec-2019 | 30-Mar-2021 |
| 380-4458-15236 | Malaysia | 1-Mar-2019 | 30-Mar-2021 |
| 380-4458-15237 | Malaysia | 1-Mar-2019 | 30-Mar-2021 |
| 380-4458-15238 | Malaysia | 1-Apr-2019 | 30-Mar-2021 |
| 380-4458-15239 | Malaysia | 1-Mar-2019 | 30-Mar-2021 |
| 380-4458-15240 | Malaysia | 18-Dec-2018 | 30-Mar-2021 |
| 380-4458-15241 | Malaysia | 24-Jan-2019 | 30-Mar-2021 |
| 380-4458-15242 | Malaysia | 26-Dec-2018 | 30-Mar-2021 |
| 380-4458-15244 | Malaysia | 1-Sep-2019 | 30-Mar-2021 |
| 380-4458-15295 | Malaysia | 3-May-2019 | 30-Mar-2021 |
| 380-4458-1549 | Puerto Rico | 10-Sep-2019 | 30-Mar-2021 |
| 380-4458-15385 | Singapore | 1-Mar-2019 | 22-Dec-2020 |
| 380-4458-14272 | South Korea | 3-Jul-2019 | 20-Oct-2020 |
| 380-4458-14285 | South Korea | 3-Dec-2019 | 6-Aug-2020 |
| 380-4458-14270 | South Korea | 13-Dec-2018 | 25-May-2020 |
| 380-4458-15252 | South Korea | 19-Apr-2019 | 30-Mar-2021 |
| 380-4458-15388 | South Korea | 28-May-2019 | 5-Mar-2021 |
| 380-4458-14276 | South Korea | 4-Dec-2019 | 1-Jun-2020 |
| 380-4458-15748 | Spain | 26-Dec-2019 | 30-Mar-2021 |
| 380-4458-2795 | Spain | 29-Oct-2018 | 28-Oct-2019 |
| 380-4458-15265 | Spain | 31-Oct-2018 | 30-Mar-2021 |
| 380-4458-15267 | Spain | 8-Mar-2019 | 30-Mar-2021 |
| 380-4458-14601 | Spain | 4-Oct-2018 | 30-Mar-2021 |
| 380-4458-15270 | Spain | 23-Jan-2019 | 28-Sep-2020 |
| 380-4458-14898 | Spain | 13-Feb-2019 | 30-Mar-2021 |
| 380-4458-2794 | Spain | 30-Oct-2018 | 30-Mar-2021 |
| 380-4458-1029 | Spain | 28-Sep-2018 | 2-Feb-2021 |
| 380-4458-2768 | Spain | 13-Feb-2019 | 30-Mar-2021 |
| 380-4458-15245 | Taiwan | 28-May-2020 | 30-Mar-2021 |
| 380-4458-15248 | Taiwan | 16-Oct-2019 | 30-Mar-2021 |
| 380-4458-14903 | Taiwan | 12-Apr-2018 | 30-Mar-2021 |
| 380-4458-15249 | Taiwan | 2-Dec-2019 | 30-Mar-2021 |
| 380-4458-15250 | Taiwan | 1-Sep-2019 | 30-Mar-2021 |
| 380-4458-15251 | Taiwan | 13-Mar-2019 | 30-Mar-2021 |
| 380-4458-15247 | Taiwan | 14-Dec-2018 | 30-Mar-2021 |
| 380-4458-15376 | Taiwan | 4-Oct-2020 | 30-Mar-2021 |
| 380-4458-15381 | Taiwan | 23-Aug-2019 | 19-Mar-2021 |
| 380-4458-6918 | Thailand | 13-Nov-2018 | 30-Mar-2021 |
| 380-4458-8033 | Thailand | 1-Apr-2019 | 30-Mar-2021 |
| 380-4458-8217 | Thailand | 1-Feb-2019 | 30-Mar-2021 |
| 380-4458-4127 | Thailand | 1-Mar-2019 | 30-Mar-2021 |
| 380-4458-4169 | Thailand | 22-Jan-2019 | 30-Mar-2021 |
| 380-4458-1936 | Thailand | 14-Nov-2018 | 30-Mar-2021 |
| 380-4458-15735 | Thailand | 8-Dec-2020 | 30-Mar-2021 |
| 380-4458-5605 | Turkey | 22-Aug-2019 | 30-Mar-2021 |
| 380-4458-3541 | Turkey | 4-Jul-2019 | 30-Mar-2021 |
| 380-4458-15080 | United States | 8-Jun-2018 | 3-Jul-2019 |
| 380-4458-15083 | United States | 15-Oct-2018 | 1-Aug-2019 |
| 380-4458-1236 | United States | 8-Jun-2018 | 30-Mar-2021 |
| 380-4458-11678 | United States | 6-Jun-2018 | 28-Aug-2019 |
| 380-4458-1534 | United States | 25-Sep-2018 | 24-Jun-2019 |
| 380-4458-1624 | United States | 6-Jun-2018 | 25-Jul-2019 |
| 380-4458-2000 | United States | 3-Dec-2018 | 29-Jul-2020 |
| 380-4458-1198 | United States | 18-Jul-2018 | 23-Aug-2019 |
| 380-4458-2843 | United States | 21-Jun-2018 | 21-Aug-2020 |
| 380-4458-2106 | United States | 12-Jun-2018 | 30-Mar-2021 |
| 380-4458-109 | United States | 6-Dec-2018 | 10-Jul-2019 |
| 380-4458-754 | United States | 7-Jun-2018 | 3-Jul-2019 |
| 380-4458-2480 | United States | 3-Jul-2018 | 31-Jul-2019 |
| 380-4458-589 | United States | 6-Jul-2018 | 3-Jul-2019 |
| 380-4458-1645 | United States | 6-Jul-2018 | 19-Aug-2019 |
| 380-4458-2475 | United States | 1-Jun-2018 | 30-Mar-2021 |
| 380-4458-2825 | United States | 6-Jun-2018 | 12-Jul-2019 |
| 380-4458-2675 | United States | 21-Jun-2018 | 31-Jul-2019 |
| 380-4458-991 | United States | 6-Aug-2018 | 4-Nov-2019 |
| 380-4458-255 | United States | 27-Jun-2018 | 15-Jun-2019 |
| 380-4458-1966 | United States | 25-May-2018 | 12-Jul-2019 |
| 380-4458-1950 | United States | 21-Aug-2019 | 30-Mar-2021 |
| 380-4458-13183 | United States | 11-Mar-2019 | 29-Sep-2020 |

Table S2. Institutional review boards.

| **Geographic Region/Country or Region Principal Investigator (Site Number)** | **Geographic Region/Country or Region** |
| --- | --- |
| **Asia Pacific/China** | |
| Chen, Jun, MD (23024) | Ethics Committee of Shanghai Public Health Clinical Centre, Shanghai, China |
| Dai, Lili, PhD (15762) | The Ethics Committee of Beijing YouAn Hospital, Capital Medical University, Beijing, China |
| Geng, Wenqing, MD (15761) | Medical Ethics Committee of The First Hospital of China Medical University, Shenyang, China |
| He, Shenghua, MB (15765) | Ethics Committee of Chengdu Public Health Clinical Centre, Sichuan, China |
| Li, Linghua, MD (15763) | Medical Ethics Committee of Guangzhou Eighth, People's Hospital, Guangzhou, China |
| Li, Taisheng, PhD (10742) | Drug Clinical Trial Ethics Committee of Peking, Union Medical College Hospital, Chinese Academy, of Medical Sciences, Beijing, China |
| Su, Minghua, MM (10899) | Medical Ethics Committee of The First Affiliated Hospital of Guangxi Medical University, Guangxi, China |
| Wang, Hui, MM (15764) | The Third People's Hospital of Shenzhen, Guangdong, China |
| Wang, Min, MD (15767) | Ethics Committee of The First Hospital of Changsha, Hunan, China |
| Wang, Fusheng, MD (11551) | Medical Ethics Committee of The Fifth Medical Centre of PLA General Hospital, Beijing, China |
| Wei, Hongxia, MM (15768) | Medical Ethics Committee of The Second Hospital of Nanjing, Jiangsu, China |
| Zhu, Biao, MD (15766) | Medical Ethics Committee of The First Affiliated Hospital of Zhejiang University, Zhejiang, China |
| Zhang, Fujie, PhD (05220) | The Ethics Committee of Beijing Ditan Hospital Capital Medical University, Chaoyang District, Beijing, China |
| **Asia Pacific/ Hong Kong** | |
| Lee, Man-Po, MD (15234) | Research Ethics Committee (Kowloon  Central/Kowloon East), Kowloon, Hong Kong |
| Lui, Chung Yan Grace, Professor (15235) | The Joint Chinese University of Hong Kong - New Territories East Cluster Clinical Research Ethics  Committee, Shatin, Hong Kong |
| Tsang, Tak Yin Owen, Doctor (05606) | Kowloon West Cluster Research Ethics Committee, Princess Margaret Hospital, Kowloon, Hong Kong |
| **Asia Pacific/ Japan** | |
| Naito, Toshio, MD (16964) | Juntendo University Hospital Institutional Review Board, Tokyo, Japan |
| Nakajima, Hideaki, MD (16966) | Yokohama City University Hospital Institutional Review Board, Yokohama, Japan |
| Oka, Shinichi, MD (07973) | Centre Hospital of the National Centre for Global Health and Medicine Institutional Review Board, Tokyo, Japan |
| Shirano, Michinori, MD (16967) | Osaka City General Hospital Institutional Review Board, Osaka, Japan |
| Shirasaka, Takuma, MD (11589) | National Hospital Organisation Osaka National Hospital Institutional Review Board, Osaka, Japan |
| Tateyama, Masao, MD (16965) | University of the Ryukyus Hospital Institutional Review Board, Okinawa, Japan |
| Yokomaku, Yoshiyuki, MD (16753) | National Hospital Organisation Nagoya Medical Centre Institutional Review Board, Nagoya, Japan |
| Yoshida, Masaki, MD (16755) | The Jikei University Hospital Institutional Review Board for Drugs, Tokyo, Japan |
| **Asia Pacific/ South Korea** | |
| Choi, Jae-phil, PhD (14276) | Seoul Medical Centre, Institutional Review Board, Seoul, South Korea |
| Choi, Jun Yong, PhD (14285) | Severance Hospital, Yonsei University Health System, Institutional Review Board, Seoul, South Korea |
| Kim, Sang-II, PhD (15252) | The Catholic University of Korea, Seoul St. Mary's Hospital Institutional Review Board, Seoul, South Korea |
| Kim, Shin-Woo, PhD (14272) | Kyungpook National University Hospital  Institutional Review Board, Daegu, South Korea |
| Kim, Yeon Sook, PhD (14270) | Chungnam National University Hospital Institutional Review Board, Daejeon, South Korea |
| Lee, Sunhee, PhD (15388) | Pusan National University Hospital Institutional Review Board, Busan, South Korea |
| **Asia Pacific/ Malaysia** |  |
| Ab Rahman, Ahmad Kashfi Bin Hj, MD (15237)  Chidambaram, Suresh Kumar, MD (15242)  Chow, Ting Soo, MD (15240)  Chua, Hock Hin, MD (15239)  Ker, Hong Bee, MD (15241)  Lee, Heng Gee, MD (15236)  Leong, Chee Loon, MD (15244)  Mat Jelani, Anilawati, MD (15238) | Medical Research and Ethics Committee (MREC), National Institutes of Health, Ministry of Health  Malaysia, Shah Alam, Malaysia |
| Syed Omar, Sharifah Faridah, MD (15295) | UMMC Medical Research Ethics Committee, University Malays Medical Centre, Kuala Lumpur, Malaysia |
| **Asia Pacific/ Singapore** | |
| Olszyna, Dariusz, MD (15385) | Domain Specific Review Board, Singapore |
| **Asia Pacific/ Taiwan** | |
| Chen, Su-Jung, PhD (15381) | Institutional Review Board, Taipei Veterans General Hospital, Taipei, Taiwan |
| Cheng, Shu-Hsing, PhD (15250) | Medical Ethics and Institutional Review Board of Taoyuan General Hospital, Taoyuan, Taiwan |
| Hung, Chien-Ching, PhD (14903) | Research Ethics Committee, National Taiwan University Hospital, Taipei, Taiwan |
| Ko, Wen-Chien, MD (15248) | Institutional Review Board National Cheng Kung University Hospital, Tainan, Taiwan |
| Lin, Yu-Hui, MD (15376) | The Institutional Review Board of Taichung Veterans General Hospital, Taichung, Taiwan |
| Lu, Po-Liang, PhD (15247) | Institution Review Board, Kaohsiung Medical University Chung-Ho Memorial Hospital No. 100, Kaohsiung, Taiwan |
| Tsai, Hung-Chin, PhD (15251) | Kaohsiung Veterans General Hospital Institution Review Board, Kaohsiung, Taiwan |
| Wang, Chien-Chun, MD (15245) | Taipei City Hospital Research Ethics Committee No.10, Taipei, Taiwan |
| Yang, Chia-Jui, MD (15249) | Research Ethics Review Committee Far Eastern Memorial Hospital, New Taipei, Taiwan |
| **Asia Pacific/ Thailand** | |
| Avihingsanon, Anchalee, PhD (01936) | Institutional Review Board, Faculty of Medicine, Chulalongkorn University, Bangkok, Thailand |
| Chetchotisakd, Ploenchan, MD (04127) | Centre for Ethics in Human Research, Khon Kaen University, Khon Kaen, Thailand |
| Khusuwan, Suwimon, MD (15735) | Central Research Ethics Committee (CREC), Bangkok, Thailand; Ethics Committee in Human Research, Chiangrai Prachanukroh Hospital, Chiang Rai, Thailand |
| Kiertiburanakul, Sasisopin, MD (08033) | The Committee on Human Rights Related to Researchers Involving Human Subjects, Ramathibodi Hospital, Bangkok, Thailand |
| Ratanasuwan, Winai, MD (06918) | Siriraj Institutional Review Board, Bangkok, Thailand |
| Siripassom, Krittaecho, MD (08217) | The Institutional Review Board of Bamrasnaradura Infectious Diseases Institute, Nonthaburi, Thailand |
| Supparatpinyo, Khuanchai, MD (04169) | Research Ethics Committee, Chiang Mai University, Chiang Mai, Thailand |
| **EMEA/ France** | |
| Aumaitre, Hugues, MD (02447)  Miailhes, Patrick, MD (02866) | CHRU Tours - Hopsital Bretonneau - CPP Ouest I, Tours, France |
| **EMEA/ Greece** | |
| Chini, Maria, MD (15266)  Metallidis, Symeon, MD (15269)  Papastamopoulos, Vasileios, MD (15272)  Psichogiou, Mina, MD (06171) | National Ethics Committee, Cholargos, Greece |
| **EMEA/ Spain** | |
| Aleman, Maria Remedios, MD, PhD (15748)  Estrada Perez, Vicente, MD, PhD (02795)  Garcia Deltoro, Miguel, MD (15265)  Garcia Garcia, Josefina, MD, PhD (15267)  Gorgolas Hernandez-Mora, Miguel, MD, PhD  (02768)  Laguno Centeno, Montserrat, MD (14898)  Martin Carbonero, Luz, MD, PhD (14601)  Miralles Álvarez, Celia, MD (02794)  Pulido Ortega, Federico, MD (01029)  Ryan Murua, Pablo, MD (15270) | CEIC Hospital Clinico San Carlos, Madrid, Spain |
| **Europe/ Turkey** | |
| Korten, Volkan, MD (03541)  Tabak, Omer Fehmi, MD (05605) | Istanbul University Cerrahpasa Medical Faculty Clinical Research Ethics Committee, Istanbul, Turkey |
| **Latin America/Dominican Republic** | |
| Koenig, Ellen Levy, MD (00986) | Asociación de Bioética, Estudios Protocolares y Seguimientos (ABIEPROSE),Santo Domingo,  Dominican Republic |
| **North America/ United States** | |
| Benson, Paul, DO (01236)  Berhe, Mezgebe, MD, MPH (11678)  Brinson, Cynthia C., MD (01624)  Goldstein, Deborah, MD (01198)  Creticos, Catherine, MD (01645)  Crofoot Jr., Gordon E., MD (02475)  Cruickshank, Frederick A., MD (02825)  Kinder, Clifford A., MD (02675)  Mayer, Cynthia A., DO (02843)  Osiyemi, Olayemi, MD (02106)  Prelutsky, David J., MD, FACP, AAHIVS (01966)  Ramgopal, Moti, MD (01950)  Rolle, Charlotte-Paige, MD, MPH (15080)  Santiago Colon, Lizette, MD (01549)  Vanig, Thanes, MD (00754)  Wohlfeiler, Michael B., MD (02480) | Advarra Institutional Review Board, Columbia, Maryland, USA |
| Brar, Indira, MD (01534) | Henry Ford Health System Institutional Review Board, Detroit, Michigan, USA |
| Fichtenbaum, Carl, MD (02000) | University of Cincinnati Institutional Review Board, Cincinnati, Ohio, USA |
| Morano, Jamie, MD, MPH (15083) | University of South Florida Institutional Review Board, Tampa, Florida, USA |
| Ogbuagu, Onyema, MD (13183) | Yale University Human Research Protection Program Human Investigation Committee, New Haven, Connecticut, USA |
| Pollard, Richard B., MD (00109) | UC Davis Institutional Review Board  Administration, Sacramento, California, USA |
| Roberts, Afsoon, MD (00589) | Western Institutional Review Board, Puyallup, Washington, USA |
| Stephens, Jeffrey, MD (00991) | Mercer University Institutional Review Board, Macon, Georgia, USA |
| Thompson, Melanie A., MD (00255) | AIDS Research Consortium of Atlanta Institutional Review Board, Atlanta, Georgia, USA |

Fig S1. Study design.

^a^Change in ALT concentration from >ULN (female participants: 25 U/mL; male participants: 35 U/mL) at baseline to ≤ULN at Week 144.

^b^Safety was assessed through the end of study.

AASLD 2018, American Association for the Study of Liver Diseases 2018 criteria; AE, adverse event; ALT, alanine aminotransferase;
B/F/TAF, bictegravir/emtricitabine/tenofovir alafenamide; c, copies; CD4, cluster of differentiation 4; DTG, dolutegravir; eGFR_CG_, estimated glomerular filtration rate by Cockcroft-Gault equation; F/TDF, emtricitabine/tenofovir disoproxil fumarate; FTC, emtricitabine; HBeAg, HBV envelope antigen; HBsAg, HBV surface antigen; HBV, hepatitis B virus; M = E, missing = excluded; OLE, open-label extension; QD, once daily; TEAE, treatment-emergent adverse event;
TFV, tenofovir; ULN, upper limit of normal.

Fig S2. Participant disposition.

B/F/TAF, bictegravir, emtricitabine, and tenofovir alafenamide; DTG, dolutegravir; F/TDF, emtricitabine and tenofovir disoproxil fumarate.

Fig S3. Median change in CD4 cell count from study baseline at OLE Week 48.

^a^Participants with CD4 count at baseline and 48 Weeks

B/F/TAF, bictegravir, emtricitabine, and tenofovir alafenamide; CD, cluster of differentiation; DTG, dolutegravir, F/TDF, emtricitabine and tenofovir disoproxil fumarate; Q, quartile.

Fig S4. Participants with normal ALT from B/F/TAF initiation (2018 AASLD criteria).

M=E analysis in the all B/F/TAF full analysis set. Based on the 2018 AASLD criteria; ULN is 25 U/L for females and 35 U/L for males. The continuous B/F/TAF and switch groups are not directly comparable due to differences in participant populations and duration of treatment.

AASLD, American Association for the Study of Liver Diseases; ALT, alanine aminotransferase; B/F/TAF, bictegravir, emtricitabine, and tenofovir alafenamide; DTG, dolutegravir; F/TDF, emtricitabine and tenofovir disoproxil fumarate; M=E, missing = excluded; ULN, upper limit of normal.

Fig S5. Fasting lipids and glucose, and weight from B/F/TAF initiation.

The continuous B/F/TAF and switch groups are not directly comparable due to differences in participant populations and duration of treatment.

B/F/TAF, bictegravir, emtricitabine, and tenofovir alafenamide; DTG, dolutegravir; F/TDF, emtricitabine and tenofovir disoproxil fumarate;
HDL, high-density lipoprotein; LDL, low-density lipoprotein; OLE, open-label extension.

Fig S6. eGFR_CG_ over time from B/F/TAF initiation.

The continuous B/F/TAF and switch groups are not directly comparable due to differences in participant populations and duration of treatment.

B/F/TAF, bictegravir, emtricitabine, and tenofovir alafenamide; eGFR_CG_, estimated glomerular
filtration rate by Cockcroft-Gault equation; DTG, dolutegravir, F/TDF, emtricitabine and tenofovir disoproxil fumarate.
